# Early Signs of Locomotor Ataxia

**Published:** 1897-02

**Authors:** 


### EARLY SIGNS OF LOCOMOTOR ATAXIA.

Professor Fournier (Practitioner, Cincinnati Lanc.-Clin.) classifies the first symptoms of locomotor ataxia as follows:

1. Westphal's sign is well known. It consists in the abolition of the patellar tendon reflex, and is present in two-thirds of the cases.

2. Romberg's sign can thus be appreciated: The eye is an indirect regulator of motion. It helps to correct deviations in walking and maintains the equilibrium. When a patient is suspected of incipient ataxia, it will often suffice to make him close his eyes when in the erect position to verify the diagnosis. In a few instants his body will oscillate, and if the malady is somewhat advanced, he will be in danger of falling.

3. The "stairs" symptom. One of the first and most constant symptoms of incipient locomotor ataxia is the difficulty with which the patient will descend stairs. If questioned closely on the subject, he will say that at the very outset of his malady he was always afraid of falling when coming down stairs.

4. The manner in which a patient crosses his legs is often significant. In the normal state, a man when performing the act lifts one leg simply to the height necessary to pass it over the other, whereas, in the affection under consideration he lifts it much higher than necessary, describing a large segment of a circle.

5. Walking at the word of command. The patient seated is told to get up and walk instantly. After rising he will hesitate, as if he wanted to find his equilibrium before starting off. If while in motion he is told to stop short, his body, obeying the impulsion, inclines forward as if about to salute, or, on the contrary, jerks himself backward in order to resist the impulsion forward.

6. The patient is asked to stand on one leg, at first with his eyes open, afterward closed. Although man is not made for this position, yet he can balance himself pretty firmly for a little while. The ataxic will experience a great deal of difficulty, and will instinctively call to his aid his other foot, so as not to fall. If his eyes are closed, he will not be able to stand one instant, and if not held, would fall heavily to the ground.

These symptoms will frequently, not be all present but they should all be sought for in order to avoid an error which might have grave consequences.—*Medical Standard.*

